# A novel cholesterol metabolism-related ferroptosis pathway in hepatocellular carcinoma

**DOI:** 10.1007/s12672-023-00822-z

**Published:** 2024-01-08

**Authors:** Weiwei Fang, Jianyong Liu, Fanguo Zhang, Cheng Pang, Xiying Li

**Affiliations:** 1 Department of Blood Transfusion, State Key Laboratory of Molecular Oncology, National Clinical Research Center for Cancer/Cancer Hospital, Institute/University, National Cancer Center, Chinese Academy of Medical Sciences and Peking Union Medical College, No.17, Nanli Road, Pan Jia Yuan, Beijing, 100021 China; 2 Department of Urology, Beijing Hospital, National Center of Gerontology, Institute of Geriatric Medicine, Institute/University/Hospital, Chinese Academy of Medical Sciences, No.1, Dahua Road, Dong Dan, 100730 Beijing China; 3 Graduate School of Peking Union Medical College, Chinese Academy of Medical Sciences, Beijing, 100730 China; 4 Excellence Future International Consulting Co, Ltd, Beijing, 101100 China

**Keywords:** Hepatocellular carcinoma, Cholesterol, Ferroptosis, IL1B, Indomethacin

## Abstract

**Background:**

Emerging studies have reported the contribution of cholesterol to hepatocellular carcinoma (HCC) progression. However, the specific role and mechanism of cholesterol metabolism on spontaneous and progressive HCC development from the point of view of ferroptosis are still worth exploring. The present study aimed to reveal a novel mechanism of cholesterol metabolism-related ferroptosis in hepatocellular carcinoma cells.

**Methods:**

Two microarray datasets (GSE25097, GSE22058) related to HCC were downloaded from Gene Expression Omnibus (GEO) datasets. Metabolomics analysis was performed by ultra performance liquid chromatography - tandem mass spectrometer (UPLC-MS/MS). The cholesterol-related proteins were downloaded from HMBD. Ferroptosis-related genes were extracted from FerrDb database. Data sets were separated into two groups. GSE25097 was used to identify ferroptosis-related genes, and GSE22058 was used to verify results. During these processes, chemical–protein interaction (CPI), protein–protein interaction (PPI), the Gene Ontology (GO), and Kyoto Encyclopedia of Genes and Genomes (KEGG) pathway enrichment analyses were conducted. Multivariate logistic regression analysis was used to test the associated pathway.

**Results:**

We identified 8 differentially expressed ferroptosis-related genes (HAMP, PTGS2, IL1B, ALOX15B, CDKN2A, RRM2, NQO1 and KIF20A) and 4 differentially expressed cholesterol-related genes (LCAT, CH25H, CEL and CYP7A1). Furthermore, based on the predicted results with STITCH, we identified indomethacin and IL1B as the essential node for cholesterol-mediated ferroptosis in hepatocellular carcinoma cell. Multivariate logistic regression analysis showed the activities of plasma IL1B in liver cancer patients enrolled have been significantly affected by the level of plasma cholesterol (P < 0.001) and the test result of IL1B is a predictor variable causing the changes of serum Fe levels (P < 0.001).

**Conclusions:**

Our findings shed new light on the association between cholesterol metabolism and ferroptosis in HCC, and suggest that IL1B is the necessary node for cholesterol to lead to ferroptosis process in HCC. Also, we identified the potential role of indomethacin in adjuvant therapy of HCC with complications of abnormal cholesterol metabolism.

**Supplementary Information:**

The online version contains supplementary material available at 10.1007/s12672-023-00822-z.

## Introduction

Hepatocellular carcinoma (HCC) represents approximately 90% of all primary liver cancers. It is an end-stage liver disease and is one of the most common reasons for cancer-related deaths [1]. Globally, HCC is the third-leading cause of cancer-related death.

HCC is asymptomatic in its early stage, which significantly delays its timely diagnosis. Those diagnosed at the advanced stage of disease are ineligible for curative surgery, and therapeutic options for advanced HCC patients are limited in availability and efficacy [2, 3]. Therefore, there is an urgent need to further understand the possible aetiological factors and new therapeutic methods to improve the prognosis of HCC patients.

Altered regulation of iron metabolism has been shown to play a role in the pathogenesis of HCC [4]. Excess intake of dietary iron can also increase the risk of HCC and iron treatment to a HCC cell line has shown to increase mesenchymal and metastatic characteristics, suggesting that high iron content may promote the development of HCC [5–7]. Ferroptosis is an iron-dependent form of regulated cell death that is driven by the lethal accumulation of lipid peroxidation [7, 8]. In recent years, a growing body of evidence supports the notion that activating ferroptosis may potently inhibit the growth of HCC cells, thus providing a scientific rationale for targeting ferroptosis as a novel therapeutic strategy for HCC. Apart from ferroptosis-inducing agents, numerous genes have also been identified as modulators or markers of ferroptosis. However, whether these ferroptosis-related genes are correlated with HCC patient prognosis remains largely unknown.

Metabolic reprogramming has become one of the important signs of cancer. Metabolic enzymes and metabolites are involved in various aspects of tumor formation. Accumulating evidence suggests that metabolites are important for HCC formation [8]. In the present study, we analyzed the associated molecular mechanisms of the ferroptosis-related genes in the development of HCC from the perspective of metabolomics.

## Materials and methods

### Patients and blood samples

The application of patient-derived materials and the protocols were approved by the Research Ethics Committee of the National Cancer Center with the approval reference number NCC2021A127 in accordance with the ethical standards as laid down in the 1964 Declaration of Helsinki and its later amendments or comparable ethical standards.

We performed a cohort design. During the same period (June 2021 to January 2022), 48 healthy controls and 48 patients with hepatocellular carcinoma from Cancer Hospital (Beijing, China) were naturally enrolled for the final validation analysis. Among them, 30 healthy controls and 30 patients with hepatocellular carcinoma were randomly selected for metabolomics analysis. The healthy control population had no comorbidities, and there was no statistically significant difference in age between the two groups by t-test (P > 0.05), but there may be a risk of false negative due to the low sample size.

### Data Collection

A total of 48 patients with liver cancer were enrolled in this study. The following variables of these patients were extracted from the medical records of the patients: the results of cholesterol and serum Fe, age, gender, tumor stage, history of prior heart disease, hypertension and diabetes mellitus. All the examining were detected in the laboratory department, Cancer hospital, Chinese Academy of Medical Sciences, by using full-automatic chemistry analyzer (Beckman Coulter AU5800). The activities of plasma IL1B were tested by Human IL1B ELISA Kit (ThermoFisher). Table 1 presents the detailed patient characteristics.

**Table 1:** Patients’ characteristics

Variables	N	%
Age, years; Median (range)	61(35–76)	
Gender		
Male	41	85.42%
Female	7	14.58%
Tumor stage		
I∼II	37	77.08%
III∼IV	11	22.92%
Prior heart disease history		
Yes	2	4.17%
No	46	95.83%
Prior hypertension history		
Yes	12	25.00%
No	36	75.00%
Prior diabetes mellitus history		
Yes	8	16.67%
No	40	83.33%

### Metabolomics analysis

The 100 µL sample was added with 300 µL cold acetonitrile and a whirlpool mixer (Scilogex MX-S) was used to shake for 30 min at room temperature, centrifuged at 12,000 rpm for 10 min at 4 ℃, and then 10 µL supernatant was detected by Ultra performance liquid chromatography - tandem mass spectrometer (UPLC-MS/MS). The MS platform included capillary high performance liquid chromatograph (Thermo Fisher scientific ultimate 3000) and mass spectrometer (AB SCIEXTM Triple TOF5600+). The positive and negative ion modes of electrospray ionization (ESI) were used for detection. ESI Source conditions are as follows: Ion Source Gas1 (Gas 1): 50, Ion Source Gas2 (Gas 2): 50, Curtain Gas (CUR): 25, Source Temperature: 500 °C (positive Ion) and 450 °C (negative ion), Ion Sapary Voltage Floating (ISVF) 5500 V (positive ion) and 4400 V (negative ion), TOF MS scan range: 100-1200Da, product ion scan range: 50-1000Da, TOF MS scan accumulation time 0.2s, product ion scan accumulation time 0.01s. Secondary mass spectra were obtained by information dependent acquisition (IDA) with high sensitivity mode and declustering potential(DP): ±60 V, Collision Energy: 35 ± 15 eV. Convert the original data obtained by LC-MS into ABF format by Analysis Base File Converter software. Import the ABF format file to MS-DIAL 4.70. Compare the extracted peak information with the database, and conduct full database retrieval of MassBank, Respect and GNPS. The three-dimensional matrix includes sample information, retention time, mass to core ratio and mass spectrum response intensity. Metabolomics analysis were provided by Beijing Biotech-Pack Scientific Co., Ltd.

### Datasets and pre-processing

Two HCC-related microarrays datasets GSE25097 and GSE22058 which are ethnically matched and both ethnic Chinese HCC patients were downloaded from GEO (Gene Expression Omnibus) database. Those datasets shared the same platform, Rosetta/Merck Human RSTA Custom Affymetrix 1.0 microarray. The mRNA expression data were processed by GEO2R, an online analysis tool based on R (Version 3.2.3). The results were downloaded and the DEGs (differentially expressed genes) were separated into two categories – i.e. the upregulated (FC > 1.5&P < 0.05) and downregulated (FC < 0.667&P < 0.05) genes in HCC patients. Clinical and expression data from TCGA-LIHC cohort was regarded as validation set. TCGA-LIHC cohort was downloaded from TCGA (https://portal.gdc.cancer.gov/).

### The ferroptosis-related genes from FerrDb and the cholesterol-related proteins from HMBD

FerrDb is the world’s first database related to ferroptosis that includes genes and substances. The ferroptosis-related genes and substances were downloaded from FerrDb [9]. The cholesterol-related proteins were downloaded from HMBD [10].

### Venn diagram

Using Venn diagram tool, the ferroptosis-related genes and substances and genes derived from the cholesterol-related proteins were selected for comparison with the DEGs identified from human HCC tissues genomic profile.

### The gene ontology and Kyoto Encyclopedia of genes and genomes enrich analysis

The selected genes were used to perform GO (Gene Ontology) analysis to determine BP (biological process), CC (cellular component), and MF (molecular function) term enrichment and perform KEGG (Kyoto Encyclopedia of Genes and Genomes pathway) enrichment. Final significant genes also underwent GO and KEGG enrichment analyses. Pathway enrichment analysis was performed for the screened metabolic substances. First, the ID of all metabolites in the KEGG database were found. Escherichia coli was selected as the background for enrichment analysis. Using KEGG metabolic pathway data as the background, topological analysis was performed to identify the possible metabolic pathways affected by biological perturbation. Furthermore, the metabolic pathways of metabolites were analyzed.

#### Gene-Gene, protein-protein and chemical–protein interaction network

GeneMANIA [11] is a website for functional analysis of genes. In this study, GeneMANIA was used to perform gene-gene interaction network.

The protein–protein interaction network was obtained from the STRING [12] database. The chemical–protein interaction network was obtained from the STITCH [13] database.

## Results

### Identification of the differentially expressed ferroptosis-related genes

**Fig. 1:**
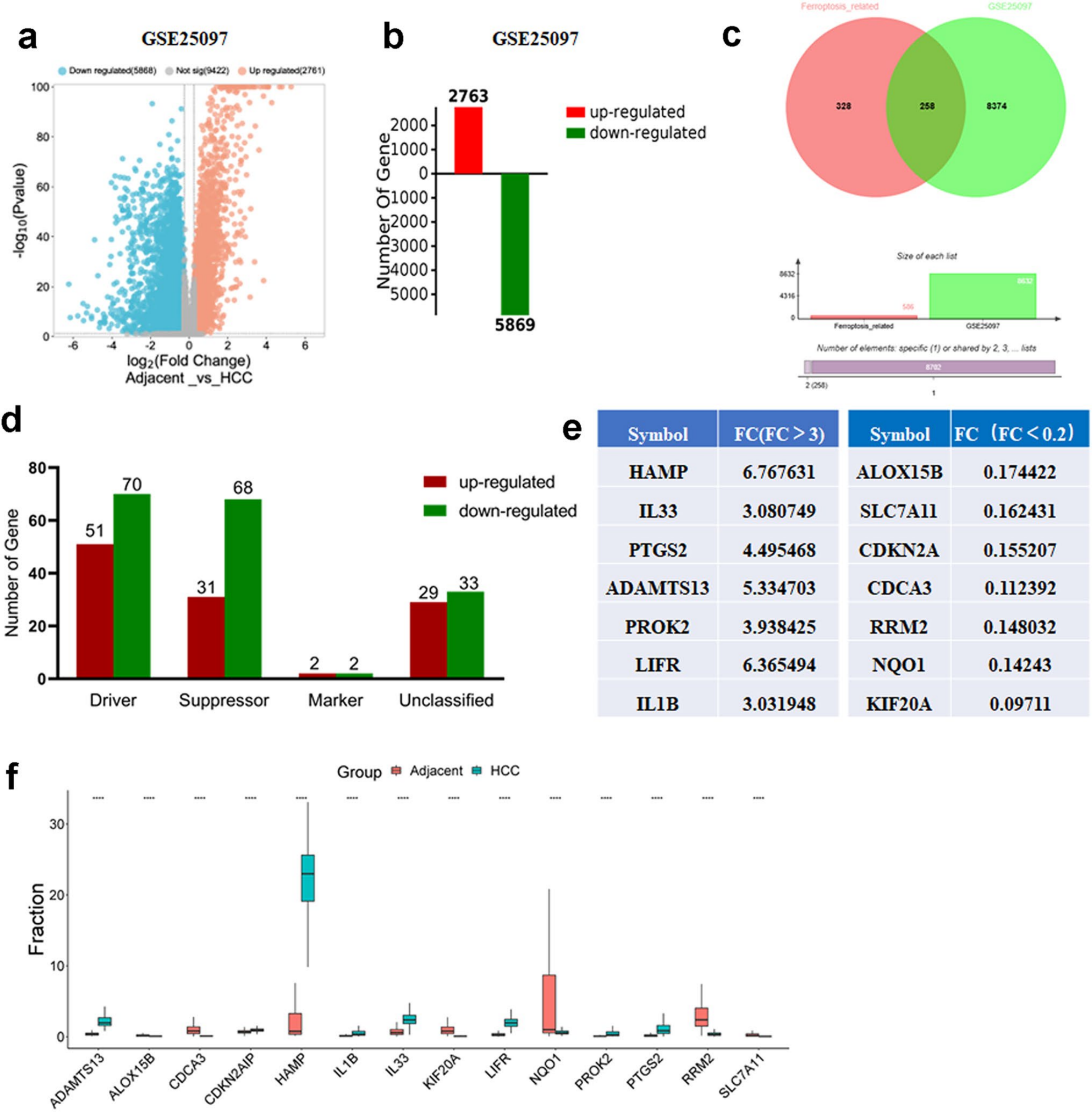
Identification of the differentially expressed ferroptosis-related genes. **a**, **b** The volcano plot and bar plot of HCC and adjacent tissues in GSE25097. **c** Venn diagram showing the intersection of ferroptosis-related DEGs. **d** The number and the categories of the 258 differentially expressed ferroptosis-related genes. The list (**e**) and the expression (**f**) of the 14 ferroptosis-related genes with the screened threshold of FC＞3 or FC＜0.2 in the 258 differentially expressed ferroptosis-related genes

The dataset, GSE25097, was downloaded from the GEO database. DEGs were screened from the GSE25097 dataset with threshold of |log2FC| > 0.58 (FC > 1.5 or FC < 0.667) and adjusted P < 0.05. Figure 1a, b shows the up-regulated and down-regulated genes in the GSE25097 dataset as volcano plots. As a result, there were 5869 down-regulated genes and 2763 up-regulated genes. The total 586 ferroptosis-related genes obtained from the FerrDb database included driver, suppressor, marker and unclassified genes. Then 258 differentially expressed ferroptosis-related genes were obtained in the intersection of the DEGs and 586 ferroptosis-related genes (Fig. 1c). The number and the categories of the 258 differentially expressed ferroptosis-related genes are shown in Fig. 1d. We further screened the 258 differentially expressed ferroptosis-related genes with threshold of FC ＞3 or FC ＜0.2. Finally, 14 ferroptosis-related genes were identified as the Focus Genes closely related to the ferroptosis pathway of HCC (Fig. 1e).

**Fig. 2:**
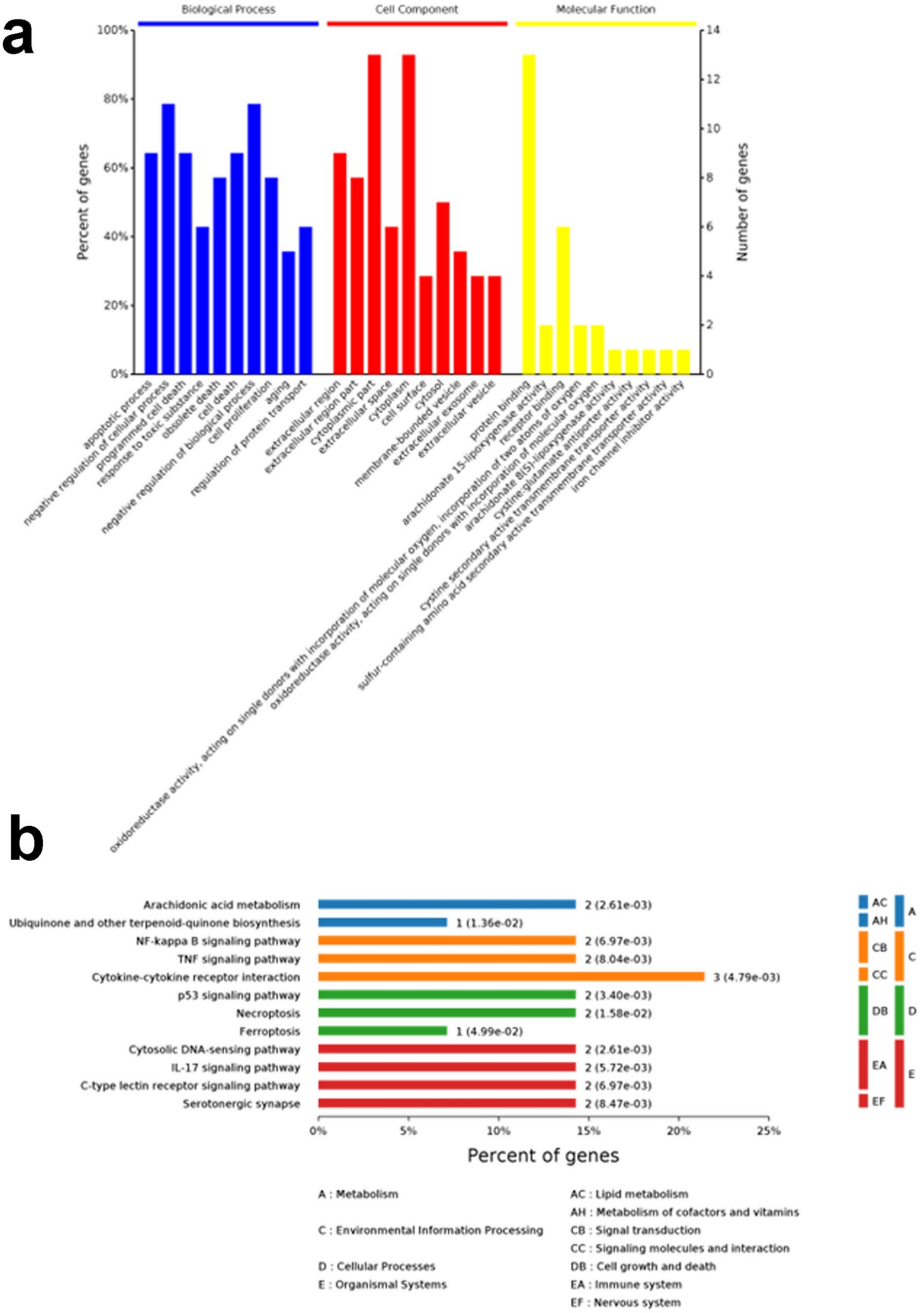
The GO and KEGG enrichment analyses using the 14 ferroptosis-related DEGs. **a** The GO functional analysis shows enriched items in the screened 14 ferroptosis-related DEGs. **b** The KEGG analysis shows the enriched items in the screened 14 ferroptosis-related DEGs

Gene Ontology term enrichment was determined for the 14 ferroptosis-related Focus Genes and included analysis for enriched BP, MF, and CC (Fig. 2a). KEGG enrichment analysis was also performed. The GO-BP results showed that these biomarkers are strongly associated with the “negative regulation of cellular process” and “negative regulation of biological process”, compared with other process. In the GO-CC, overlapping genes were mostly enriched in the “cytoplasmic part” and “cytoplasm” terms. The two most enriched terms in GO-MF were “protein binding” and “receptor binding”. Furthermore, KEGG results illustrated that these Focus Genes were mainly enriched in the “A-Metabolism”, “C-Environmental Information Process”, “D-Cellular process” and “E-Organismal Systems” pathways (Fig. 2b). Among them, the most enriched terms in KEGG were “CC-Signaling molecules and interaction”.

### Identification of the differentially ferroptosis-related metabolites

**Fig. 3:**
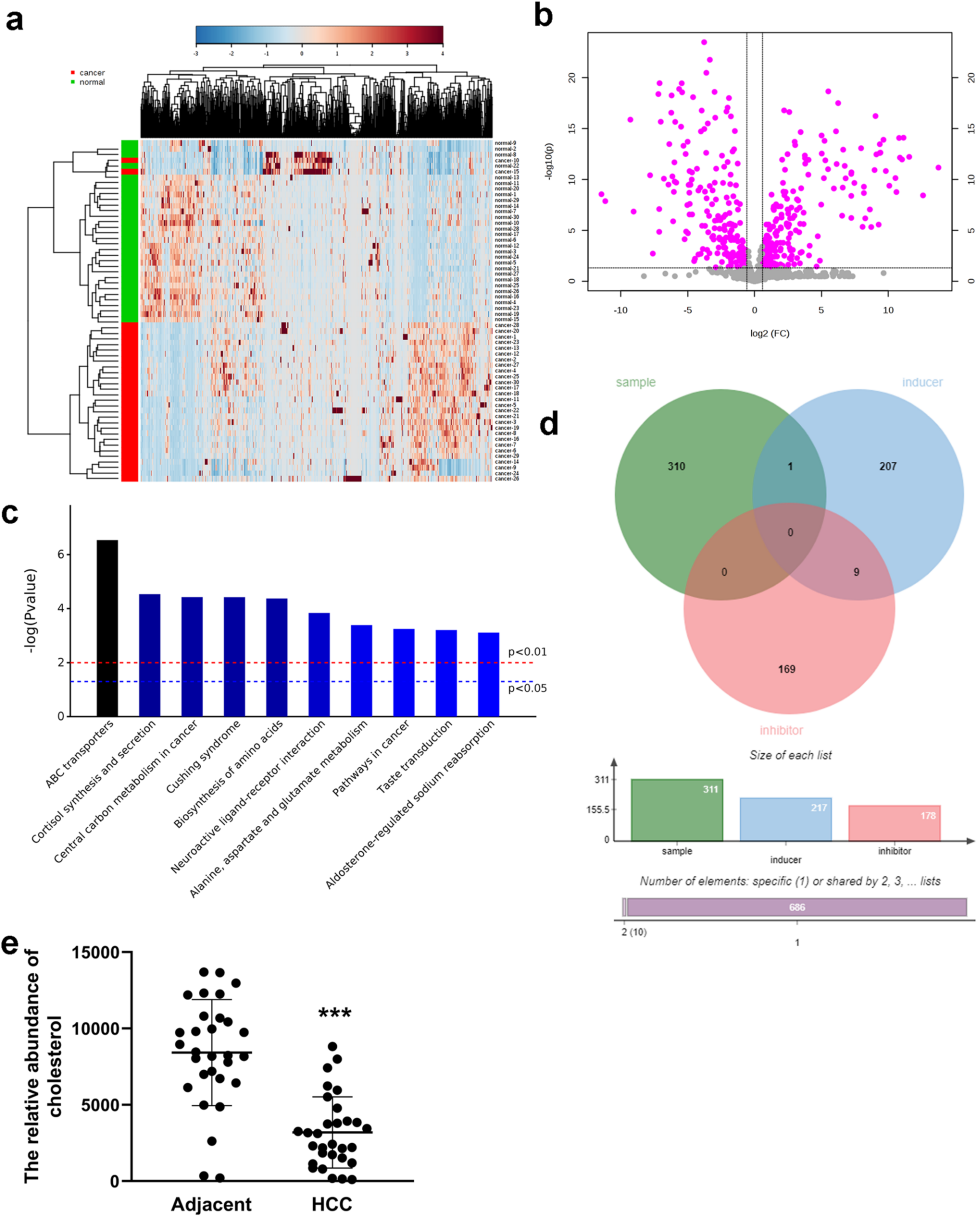
Identification of the differentially ferroptosis-related metabolites. **a**, **b** The heatmap and volcano plot of metabolomics analysis with the peripheral blood of HCC patients and the healthy controls. **c** Pathway enrichment analysis of the aberrantly regulated metabolites. **d** Venn diagram showing the union set of 311 differential metabolites and 386 ferroptosis-related substances. **e** The relative abundance of cholesterol in adjacent control and HCC group

To identify metabolites that are potentially involved in ferroptosis process of hepatic carcinoma cells, we first searched for metabolites that are upregulated or downregulated in the peripheral blood of HCC patients. With the screened threshold of |log2FC| > 0.58 (FC > 1.5 or FC < 0.667) and adjusted P < 0.05, metabolomics profiling identified 311 metabolites that were aberrantly regulated in the peripheral blood of HCC patients, compared to the peripheral blood of healthy controls (Fig. 3a, b). Pathway enrichment of the aberrantly regulated metabolites indicated ABC transporters, cortisol synthesis and secretion, central carbon metabolism in cancer, cushing syndrome, biosynthesis of amino acids and others (Fig. 3c). The total 386 ferroptosis-related substances obtained from the FerrDb database included 217 inducers and 178 inhibitors. In Fig. 3d, the Venn diagram shows the union set of 311 differential metabolites (Additional file 2: Excel S1) and 386 ferroptosis-related substances. Finally, only one ferroptosis-related substance, cholesterol, was identified as the key metabolite involved in ferroptosis process of hepatic carcinoma cells. The relative abundance of cholesterol in adjacent control and HCC group was shown in Fig. 3e. The relative abundance of cholesterol was aberrantly decreased in the liver of HCC patients, compared to the livers of the adjacent control.

### Identification of the differentially expressed cholesterol-related genes

**Fig. 4:**
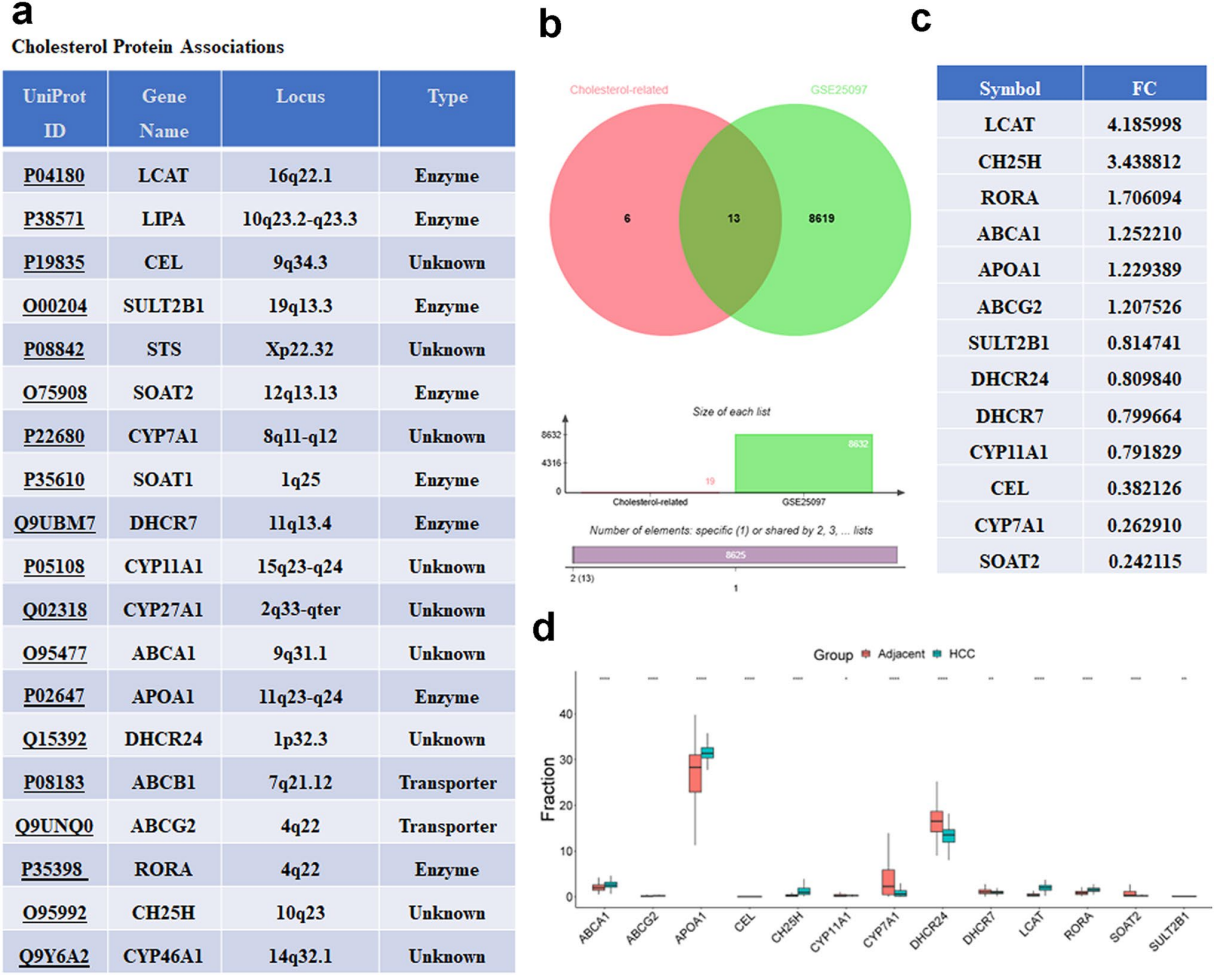
Identification of the differentially expressed cholesterol-related genes. **a** The list of the 19 cholesterol-related proteins downloaded from HMBD. **b** Venn diagram showing the intersection of the DEGs and 19 cholesterol-related genes corresponding to the 19 cholesterol-related proteins. The list (**c**) and the expression (**d**) of the 13 differentially expressed cholesterol-related genes in the intersection of the DEGs and 19 cholesterol-related genes corresponding to the 19 cholesterol-related proteins

The 19 cholesterol-related proteins were downloaded from HMBD (Fig. 4a). Then 13 differentially expressed cholesterol-related genes were obtained in the intersection of the DEGs and 19 cholesterol-related genes corresponding to the 19 cholesterol-related proteins downloaded from HMBD (Fig. 4b, c).

**Fig. 5:**
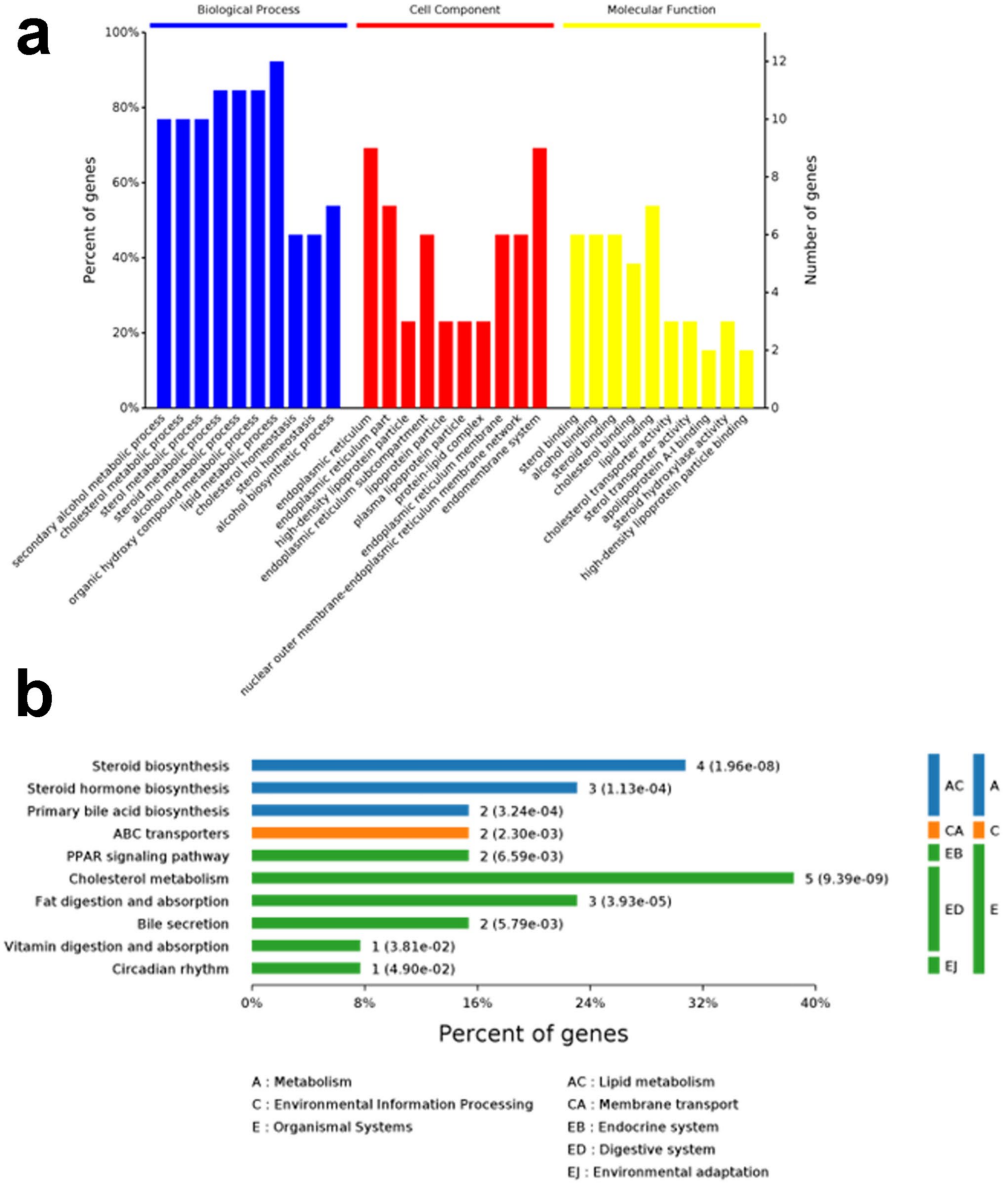
The GO and KEGG enrichment analyses using the 13 cholesterol-related DEGs. **a** The GO functional analysis shows enriched items in the screened 13 cholesterol-related DEGs. **b** The KEGG analysis shows the enriched items in the screened 13 cholesterol-related DEGs

Gene Ontology term enrichment was determined for the 13 differentially expressed cholesterol-related genes and included analysis for enriched BP, MF, and CC (Fig. 5a). KEGG enrichment analysis was also performed. The GO-BP results showed that these genes are strongly associated with the “lipid metabolic process”, “steroid metabolic process”, “alcohol metabolic process” and “organic hydroxy compound metabolic process”, compared with other process. In the GO-CC, overlapping genes were mostly enriched in the “endomembrane system” and “endoplasmic reticulum” terms. The four most enriched terms in GO-MF were “lipid binding”, “sterol binding”, “alcohol binding” and “steroid binding”. Furthermore, KEGG results illustrated that the 13 differentially expressed cholesterol-related genes were mainly enriched in the “CC- cholesterol metabolism” and “steroid biosynthesis” (Fig. 5b).

### The preliminary analysis of cholesterol metabolism-related ferroptosis pathway in hepatocellular carcinoma cells

**Fig. 6:**
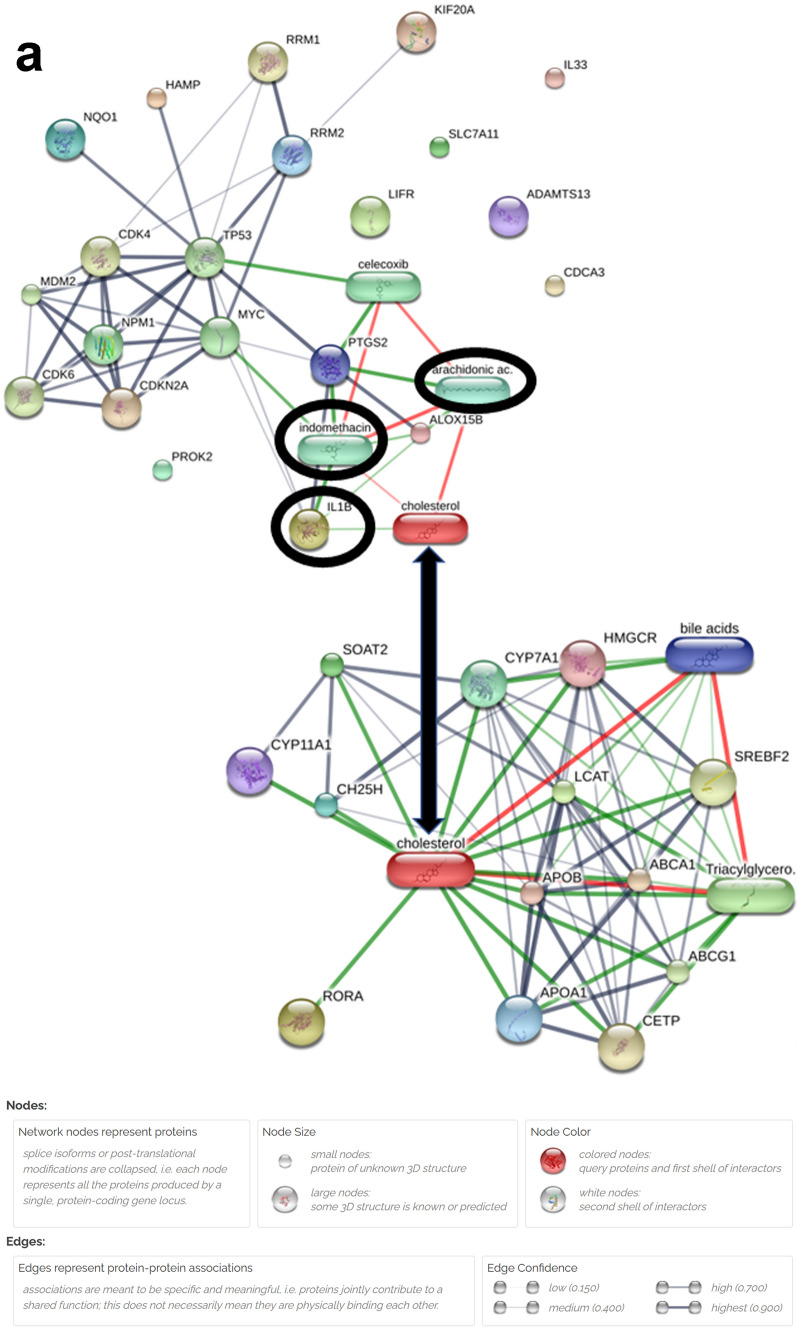
The preliminary analysis of cholesterol metabolism-related ferroptosis pathway in hepatocellular carcinoma cells. **a** The cholesterol and proteins corresponding to 14 differentially expressed ferroptosis-related genes and 13 differentially expressed cholesterol-related genes interaction network

The STITCH database was used to explore known and predicted interactions between cholesterol and proteins corresponding to 14 differentially expressed ferroptosis-related genes and 13 differentially expressed cholesterol-related genes (Fig. 6a). From the networks, we visually observed cholesterol is the bridge connecting substance metabolism and ferroptosis process in HCC. Two chemicals, arachidonic and indomethacin, and one protein, IL1B, were identified as the necessary node for cholesterol to lead to ferroptosis process.

### The verification of the overlapped genes in GSE22058 and TCGA-LIHC

**Fig. 7:**
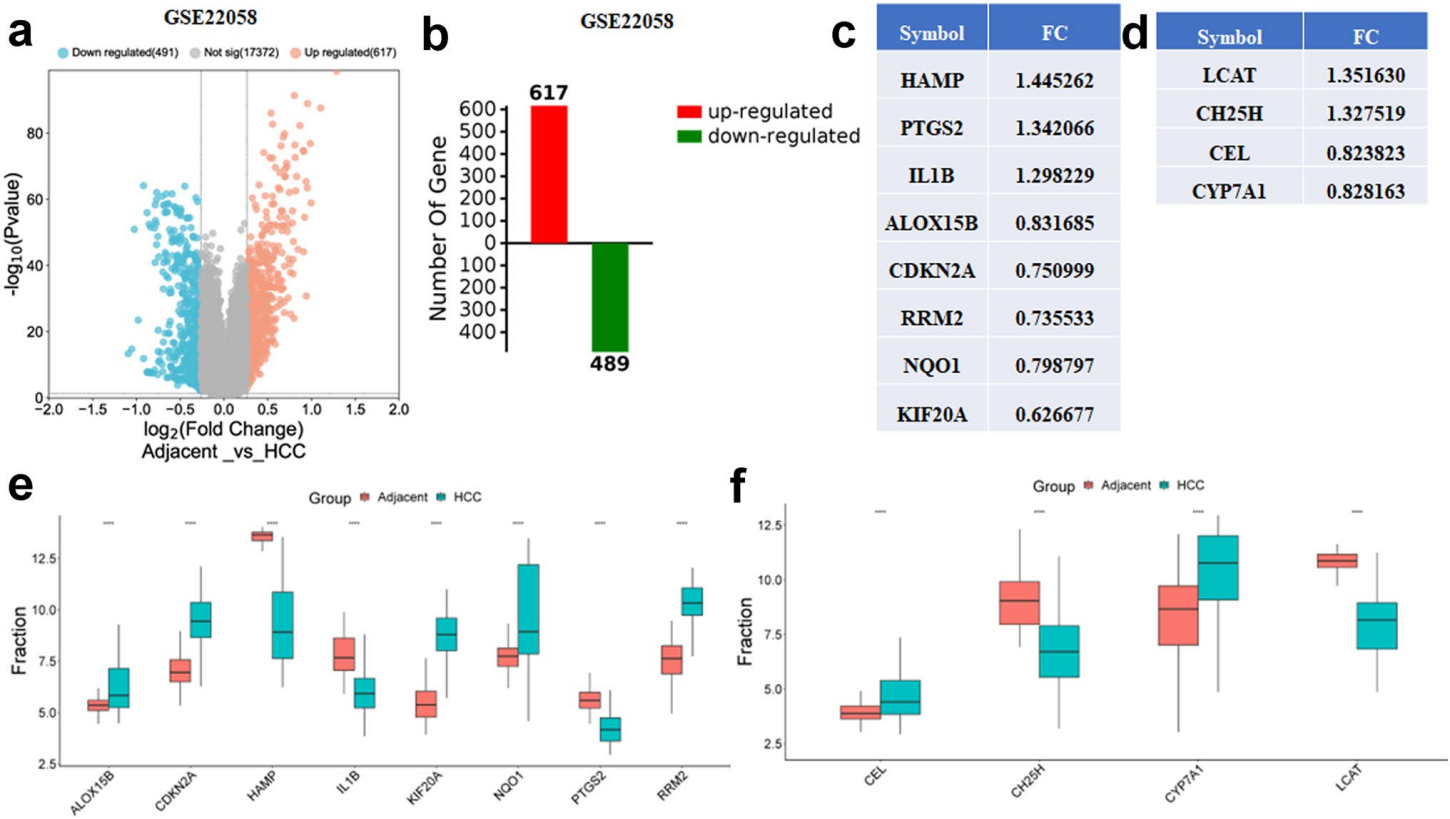
The verification of the overlapped genes in GSE22058. **a**, **b ** The volcano plot and bar plot of HCC and adjacent tissues in GSE22058. The list (**c**, **d**) and the expression of the 8 differentially expressed ferroptosis-related genes (**e**) and the 4 differentially expressed cholesterol-related genes (**f**) identified in GSE22058 based on the 14 differentially expressed ferroptosis-related genes and the 13 differentially expressed cholesterol-related genes

After selecting the 14 differentially expressed ferroptosis-related genes and the 13 differentially expressed cholesterol-related genes, the GSE22058 dataset was used to verify results. The dataset, GSE22058, was also downloaded from the GEO database. DEGs were screened from the GSE22058 dataset with threshold of |log2FC| > 0.58 (FC > 1.5 or FC < 0.667) and adjusted P < 0.05. Figure 7a, b shows the up-regulated and down-regulated genes in the GSE22058 dataset as volcano plots. As a result, there were 489 down-regulated genes and 617 up-regulated genes. Then 8 differentially expressed ferroptosis-related genes were obtained in the intersection of the DEGs and 14 differentially expressed ferroptosis-related genes identified above (Fig. 7c) and 4 differentially expressed cholesterol-related genes were obtained in the intersection of the DEGs and 13 differentially expressed cholesterol-related genes identified above (Fig. 7d). Further cox analysis with P < 0.05 and differential expression was performed in TCGA-LIHC to validate the 8 differentially expressed ferroptosis-related genes and the 4 differentially expressed cholesterol-related genes screened above (Additional file 1: Fig. S2a, b and c).

**Fig. 8:**
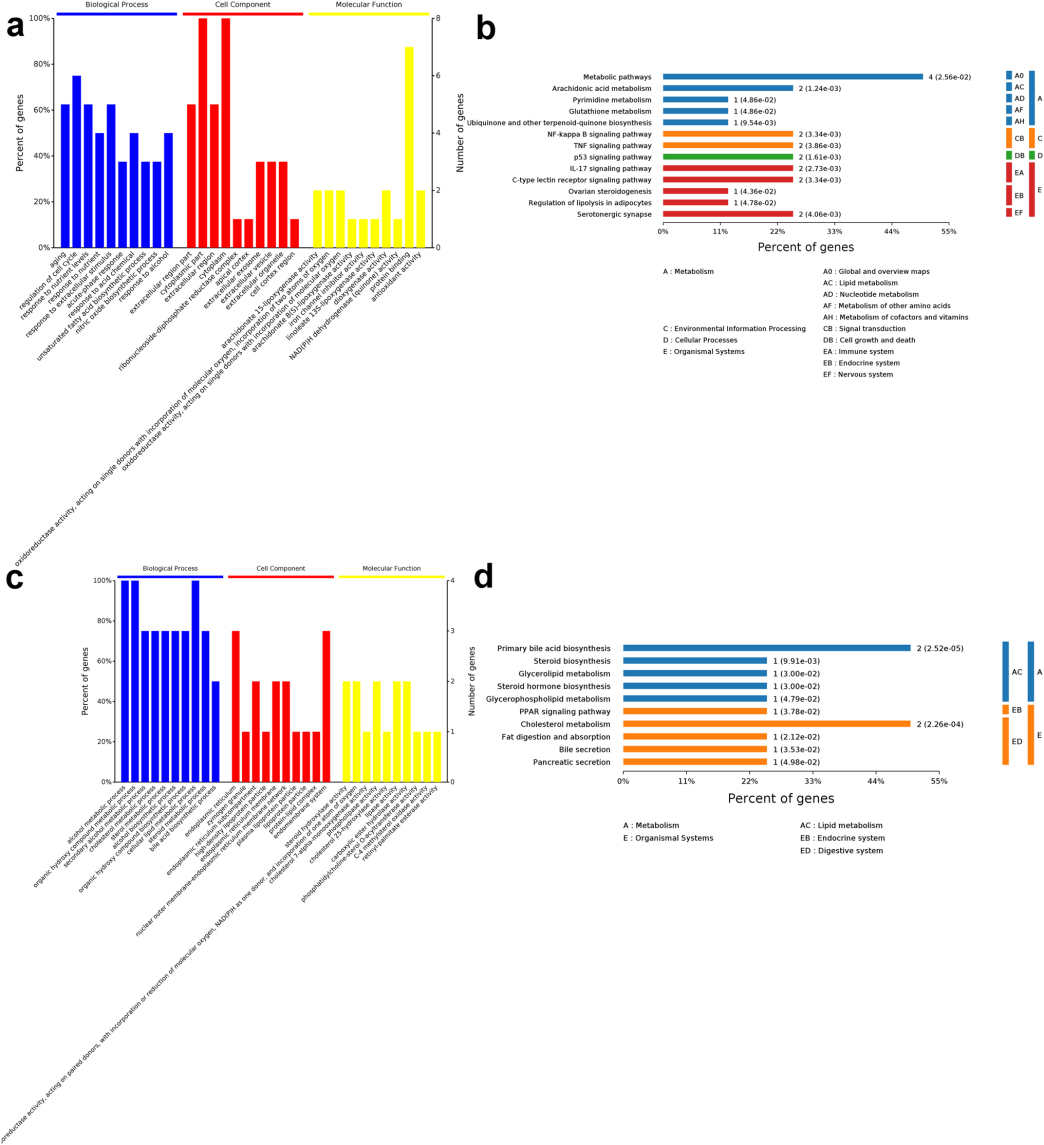
The GO and KEGG enrichment analyses using the final screened DEGs. The GO functional analysis (**a**) and the KEGG analysis (**b**) show the enriched items in the screened 8 ferroptosis-related DEGs. The GO functional analysis (**c**) and the KEGG analysis (**d**) show the enriched items in the screened 4 cholesterol-related DEGs

 GO and KEGG analysis results of these 8 differentially expressed ferroptosis-related genes are presented in Fig. 8a, b. The GO and KEGG enrichment analysis of the 4 differentially expressed cholesterol-related genes is also shown in Fig. 8c, d.

### The secondary analysis of cholesterol metabolism-related ferroptosis pathway in hepatocellular carcinoma cells

**Fig. 9:**
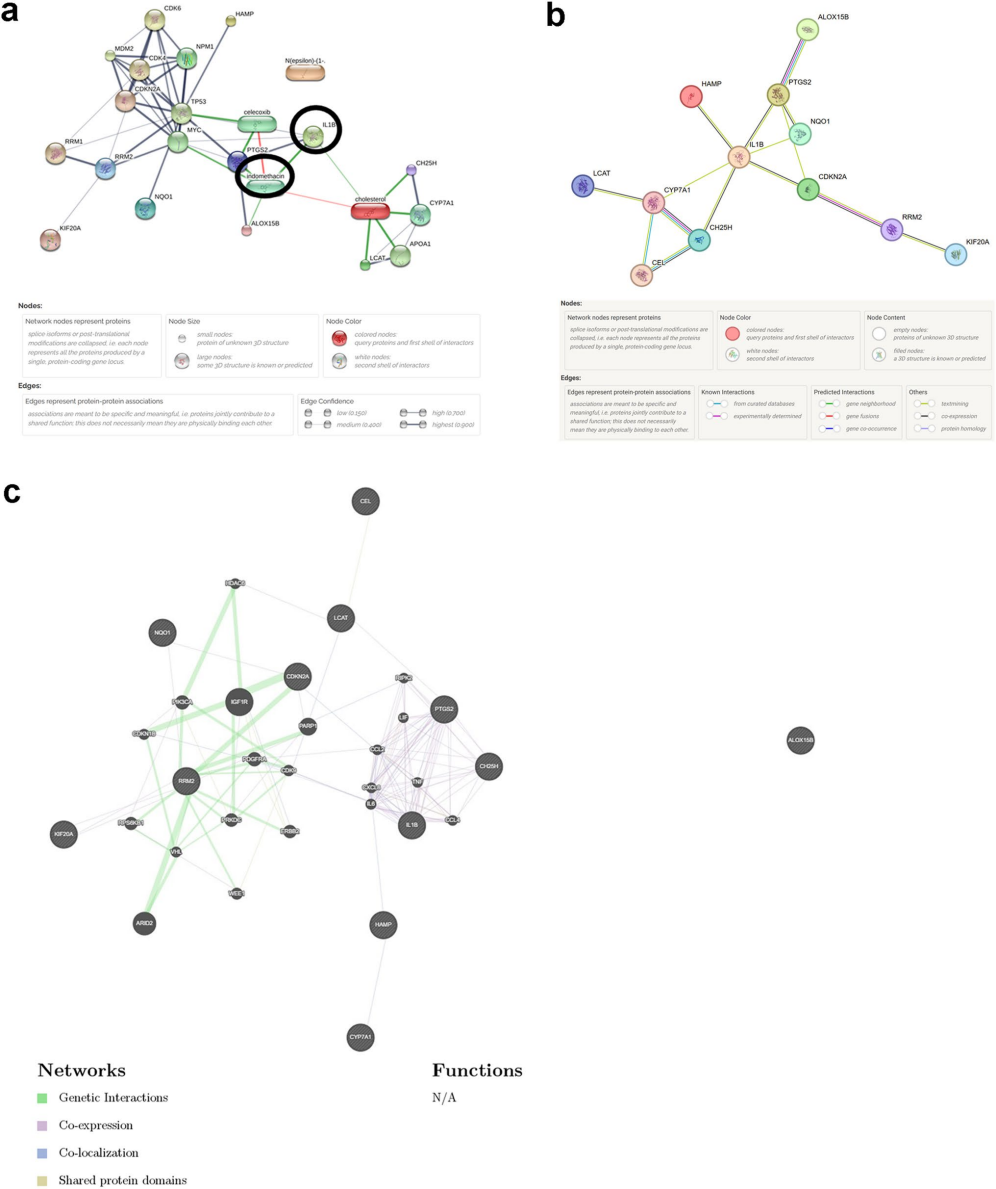
The secondary analysis of cholesterol metabolism-related ferroptosis pathway in hepatocellular carcinoma cells. **a** The cholesterol and proteins corresponding to 8 differentially expressed ferroptosis-related genes and 4 differentially expressed cholesterol-related genes interaction network. **b** The correlation network of the 8 differentially expressed ferroptosis-related proteins and 4 differentially expressed cholesterol-related proteins. **c** The correlation network of the 8 differentially expressed ferroptosis-related genes and 4 differentially expressed cholesterol-related genes

 The STITCH database was used to perform a secondary interactions’ prediction between cholesterol and proteins corresponding to 8 differentially expressed ferroptosis-related genes and 4 differentially expressed cholesterol-related genes (Fig. 9a). Finally, one chemical, indomethacin, and one protein, IL1B, were identified as the essential node for cholesterol-mediated ferroptosis in hepatocellular carcinoma cell. The correlation network of the 8 differentially expressed ferroptosis-related proteins and 4 differentially expressed cholesterol-related proteins was obtained from the STRING database (Fig. 9b). The correlation network analyzed by GeneMANIA of the 8 differentially expressed ferroptosis-related genes and 4 differentially expressed cholesterol-related genes were respectively displayed in Fig. 9c.

### Multivariate logistic regression analysis of plasma IL1B and Fe in patients with Liver cancer

Multivariate logistic regression results of the all patients enrolled associated with the changes of plasma IL1B and Fe are separately shown in Tables 2 and 3. The activities of plasma IL1B in liver cancer patients enrolled have been significantly affected by the level of plasma cholesterol (P < 0.001) and the test result of IL1B is a predictor variable causing the changes of serum Fe levels (P < 0.001). As Tables 2 and 3 showed, the influence of age, gender, tumor stage, history of prior heart disease, hypertension and diabetes mellitus were eliminated in this study.

**Table 2:** Multivariate logistic regression models examining factors associated with the changes in IL1B expression

Variable	p Value	95%CI
Age	0.876	− 0.162, 0.190
Gender	0.391	− 5.984, 2.343
Tumor stage	0.857	− 1.296, 1.558
Prior heart disease history	0.810	− 4.041, 5.169
Prior hypertension history	0.531	− 5.657, 2.917
Prior diabetes mellitus history	0.584	− 8.149, 4.592
Cholesterol	0.000	5.653, 17.314

**Table 3:** Multivariate logistic regression models examining factors associated with the changes in serum Fe expression

Variable	p Value	95%CI
Age	0.239	− 0.123, 0.031
Gender	0.248	− 2.250, 0.582
Tumor stage	0.663	− 0.454, 0.714
Prior heart disease history	0.930	− 4.041, 5.169
Prior hypertension history	0.265	− 0.666, 2.423
Prior diabetes mellitus history	0.404	− 2.627, 1.057
IL1B	0.000	0.534, 1.456

## Discussion

Globally, liver cancer is the third leading cause of cancer-related death [14]. HCC is the main type of liver cancer. Despite significant progress has been achieved in recent years, the prognosis of HCC remains poor. Therefore, it is urgent to find new strategies to guide the diagnosis and treatment of HCC.

Ferroptosis, which is an emerging type of cell death induced by metal iron and reactive oxygen species and driven by lipid peroxidation [15], is a new horizon for scientists to explore novel biomarkers and therapeutic targets [16]. A large study showed that altered regulation of iron metabolism has a strong relationship with the mechanisms involved in the pathogenesis of HCC, encouraging research into the interactions and associations between HCC and ferroptosis [17, 18]. Exploring the relationship between ferroptosis and HCC may lead to identification of new biomarkers for HCC diagnosis and new targets for HCC treatment.

Cholesterol, as a structural component of cells, has attracted more and more attention in recent years. Dietary cholesterol has an important impact on plasma and hepatic cholesterol homeostasis and cholesterol accumulation can promote the progress of HCC. A previous study showed that lncFAL stabilized by HDLBP, an important transporter that protects cells from overaccumulation of cholesterol, inhibits ferroptosis vulnerability by diminishing Trim69-dependent FSP1 degradation in hepatocellular carcinoma [19]. Although the contribution of cholesterol to HCC progression has been reported [20], the specific role and mechanism of cholesterol metabolism on spontaneous and progressive HCC development or treatment from the point of view of ferroptosis are still worth exploring. In this context, the present study aimed to reveal a novel mechanism of cholesterol metabolism-related ferroptosis in hepatocellular carcinoma cells.

In this study, we screened 14 differentially expressed ferroptosis-related genes and 13 differentially expressed cholesterol-related genes based on a systematical analysis from GEO database. Then, we explored the interactions between cholesterol and proteins corresponding to 14 differentially expressed ferroptosis-related genes and 13 differentially expressed cholesterol-related genes through venn analysis. The expression of the screened genes was validated by GSE22058 database and finally identified 8 differentially expressed ferroptosis-related genes (HAMP, PTGS2, IL1B, ALOX15B, CDKN2A, RRM2, NQO1 and KIF20A) and 4 differentially expressed cholesterol-related genes (LCAT, CH25H, CEL and CYP7A1). Further function analysis revealed that the 8 differentially expressed ferroptosis-related genes are enriched in metabolic pathways, arachidonic acid metabolism, NF-kappa B signaling pathway, TNF signaling pathway, p53 signaling pathway, IL-17 signaling pathway, C-type lectin receptor signaling pathway, serotonergic synapse and other related pathways, and the 4 differentially expressed cholesterol-related genes are enriched in primary bile acid biosynthesis, cholesterol metabolism and other related pathways.

Furthermore, based on the predicted results with STITCH, we identified one most significant small molecule drugs (indomethacin) as potential ferroptosis-related candidate drugs linked cholesterol metabolism for HCC patients and one protein (IL1B) as the essential node for cholesterol-mediated ferroptosis in hepatocellular carcinoma cell. To date, experimental and clinical studies suggest a beneficial effect from the use of indomethacin in adults with uncontrolled intracranial pressure [21]. Previous studies have found that the ability of indomethacin to inhibit the synthesis of prostaglandins when administered prophylactically may explain some of the successful results that have been reported in the prevention of cystoid macular edema [22]. We believe that this small molecule drug predicted by STITCH may be a potential ferroptosis-related drug candidate which may be suitable for the specific population of cholesterol metabolism. However, there is no evidence of the efficacy of indomethacin in the adjuvant treatment of hepatocellular carcinoma which is the one limitation of this paper. Further studies are warranted in order to determine the precise role of indomethacin in adjuvant therapy of HCC. IL1B is the gatekeeper of inflammation. It is produced mainly by immune cells, challenged by invading pathogens and danger signals and has been found to exert angiogenic functions. He et al. revealed that IL1B-induced SLC7A11 overexpression up-regulated PD-L1 and CSF1 through the αKG/HIF1α axis, which promoted TAM and MDSC infiltration, which providing a therapy direction for the inhibition of SLC7A11-mediated HCC metastasis [23]. Zong et al. found that the IL1B signaling is the underlying mechanism for the M1 macrophages to induce PD-L1 expression in HCC cells [24]. The study performed by Dang et al. showed that upregulated HOXC10 induced by IL1B promotes HCC metastasis by transactivating PDPK1 and VASP expression [25]. Li et al. found a novel regulatory molecular mechanism of caspase-1/IL1B by CD44s, which might provide potential therapeutic targets for HCC inhibition [26]. Our Multivariate logistic regression results further revealed that cholesterol, as a predictor of IL1B activity change, may play a role in ferroptosis of HCC cells by interfering with IL1B and then affecting serum iron level. Our results indicated the specific mechanism by which cholesterol metabolism regulates the ferroptosis in hepatocellular carcinoma cells through IL1B signaling. Therefore, targeting IL1B signaling is a potential new therapeutic strategy to simultaneously block angiogenesis and dampen inflammation, impairing HCC metastasis and progression. Some agents targeting IL1B, such as soluble decoy IL-1 receptor, IL-1 receptor antagonist and neutralizing monoclonal antibodies against IL-1, have been approved in clinics to treat inflammatory and autoimmune diseases diseases [24, 27, 28]. Inspired by our above study, further investigation should be carried out to observe whether these drugs may serve as adjuvants in the treatment of HCC with complications of abnormal cholesterol metabolism to induce the ferroptosis of hepatocellular carcinoma cells.

In short, we identified a novel cholesterol metabolism-related ferroptosis pathway in HCC, but the actual function of the novel pathway needs to be further proved by more trials. The other one limitation of our study is the low sample size in metabolomics study and expanding the metabolomics study will be the aim in our future study.

### Supplementary Information

**Additional file 1: Figure S1. **Analysis related to LCMS. **a** OPLS-DA model score plot. **b** PCA model score plot. **c** PLS-DA model score plot. **d** Positive ion chromatogram. **e** Negative ion chromatogram. **Figure S2. **The verification of the overlapped genes in TCGA-LIHC. **a** The volcano plot of HCC and adjacent tissues in TCGA-LIHC. The expression of the 8 differentially expressed ferroptosis-related genes (**b**) and the 4 differentially expressed cholesterol-related genes (**c**) identified in TCGA-LIHC based on the 14 differentially expressed ferroptosis-related genes and the 13 differentially expressed cholesterol-related genes.

**Additional file 2:** **Excel S1. **List of substance identified for metabolomics analysis which is the basis for all analysis results.

## Data Availability

The datasets used and/or analyzed during the current study are available from the corresponding author on reasonable request.

## Abbreviations

HCC: Hepatocellular carcinoma
GEO: Gene Expression Omnibus
UPLC-MS/MS: ultra performance liquid chromatography - tandem mass spectrometer
CPI: Chemical–protein interaction
PPI: Protein–protein interaction
GO: Gene Ontology
BP: Biological process
CC: Cellular component
MF: Molecular function
KEGG: Kyoto Encyclopedia of Genes and Genomes
DEGs: Differentially expressed genes
