# Supplementary figures and images for: A novel cholesterol metabolism-related ferroptosis pathway in hepatocellular carcinoma
Source: Discov Oncol. 2024 Jan 8;15:7. doi: 10.1007/s12672-023-00822-z (PMC10774324; doi:10.1007/s12672-023-00822-z)

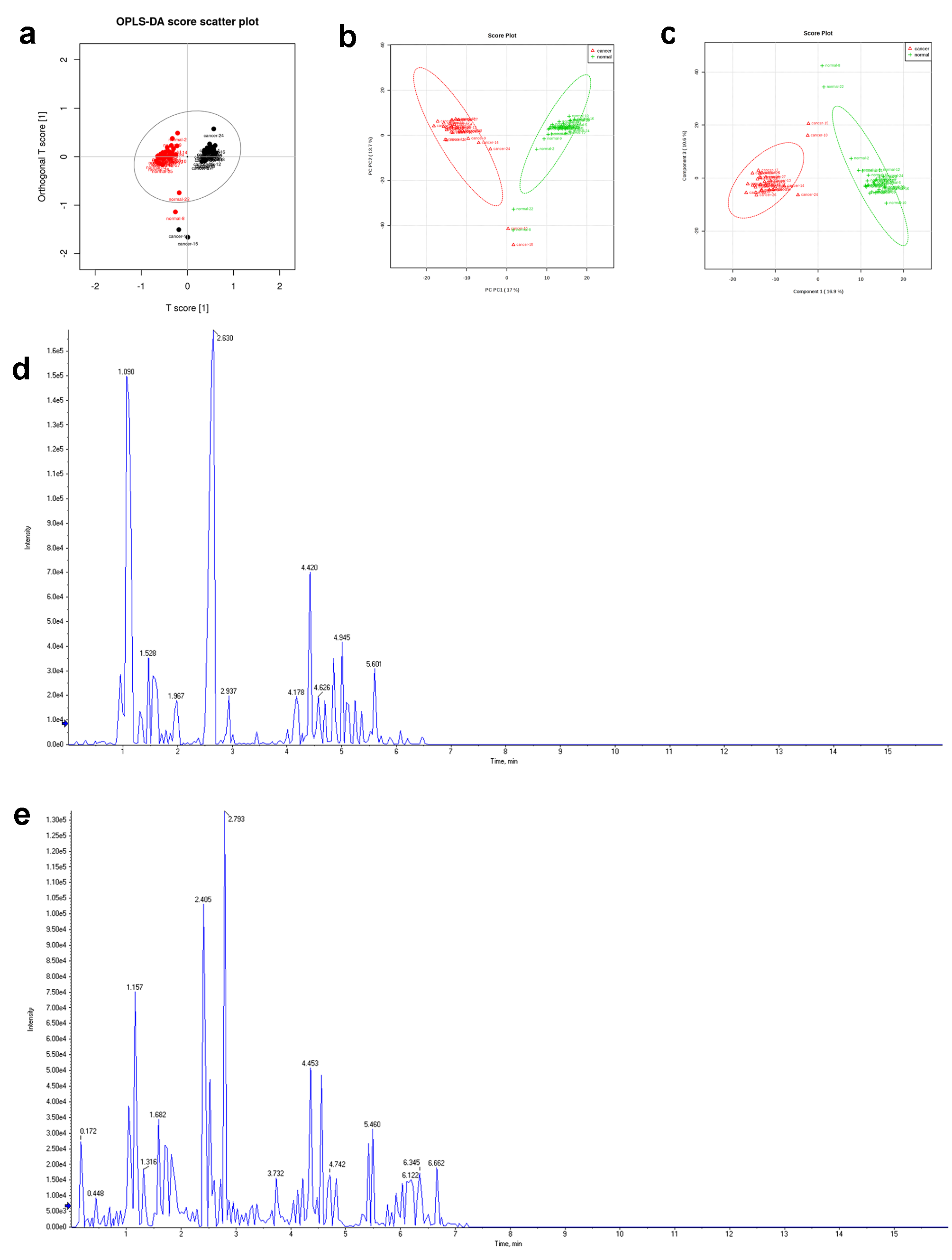

Supplement: Supplementary file 1 — Additional file 1: Figure S1. Analysis related to LCMS. a OPLS-DA model score plot. b PCA model score plot. c PLS-DA model score plot. d Positive ion chromatogram. e Negative ion chromatogram. Figure S2. The verification of the overlapped genes in TCGA-LIHC. a The volcano plot of HCC and adjacent tissues in TCGA-LIHC. The expression of the 8 differentially expressed ferroptosis-related genes (b) and the 4 differentially expressed cholesterol-related genes (c) identified in TCGA-LIHC based on the 14 differentially expressed ferroptosis-related genes and the 13 differentially expressed cholesterol-related genes. [file 12672_2023_822_MOESM1_ESM.zip › Fig S1/SFig 1.tif]

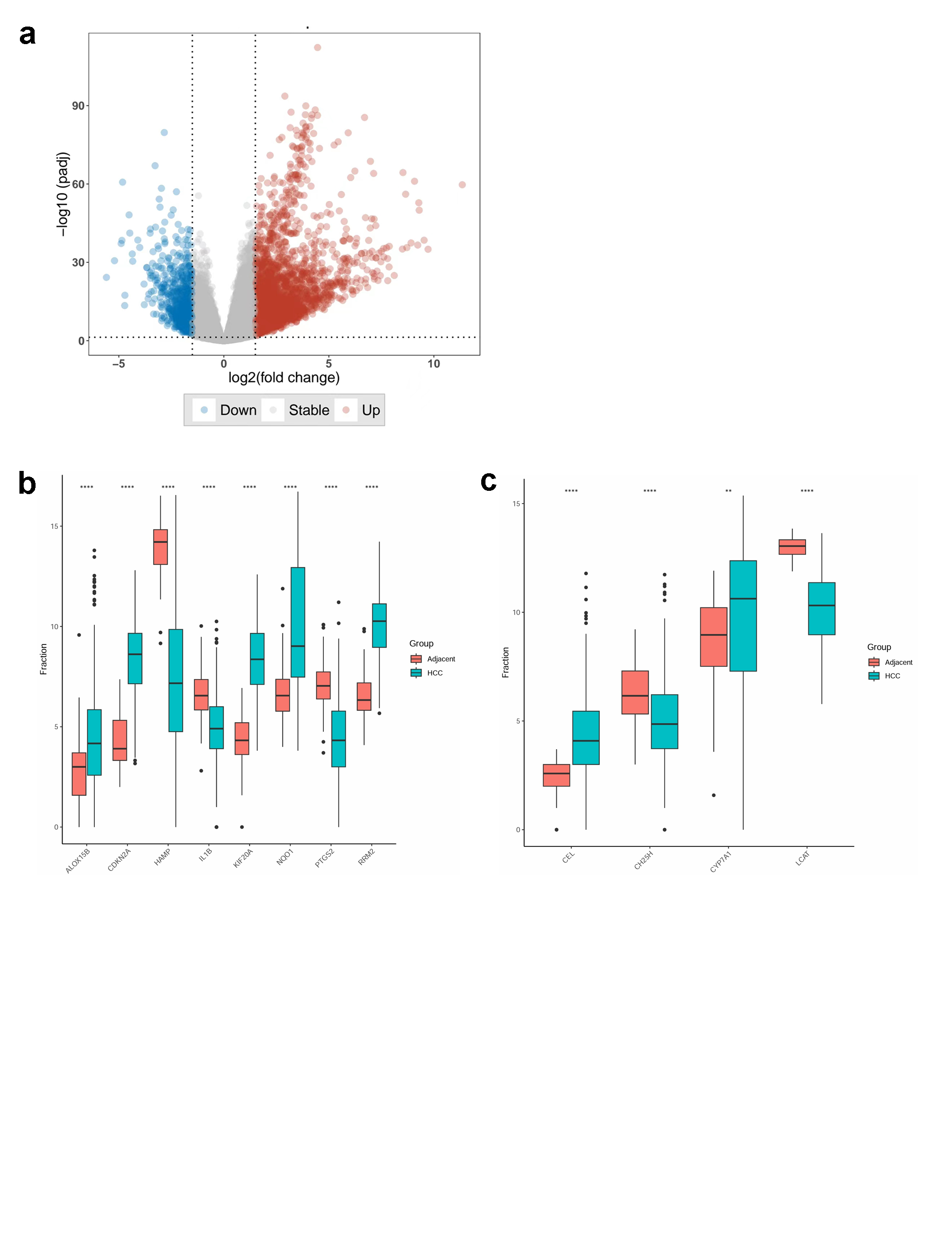

Supplement: Supplementary file 1 — Additional file 1: Figure S1. Analysis related to LCMS. a OPLS-DA model score plot. b PCA model score plot. c PLS-DA model score plot. d Positive ion chromatogram. e Negative ion chromatogram. Figure S2. The verification of the overlapped genes in TCGA-LIHC. a The volcano plot of HCC and adjacent tissues in TCGA-LIHC. The expression of the 8 differentially expressed ferroptosis-related genes (b) and the 4 differentially expressed cholesterol-related genes (c) identified in TCGA-LIHC based on the 14 differentially expressed ferroptosis-related genes and the 13 differentially expressed cholesterol-related genes. [file 12672_2023_822_MOESM1_ESM.zip › Fig S1/SFig 2.tif]
